# Effect of air velocity, temperature, and relative humidity on drying kinetics of rubberwood

**DOI:** 10.1016/j.heliyon.2020.e05151

**Published:** 2020-10-08

**Authors:** Malisa Chanpet, Nirattisai Rakmak, Nirundorn Matan, Chairat Siripatana

**Affiliations:** a School of Engineering and Technology, Walailak University, 80161, Nakhon Si Thammarat, Thailand; b Biomass and Oil-Palm Excellence Center, Walailak University, 80161, Nakhon Si Thammarat, Thailand

**Keywords:** Mechanical engineering, Thermodynamics, Kiln drying, Drying kinetics, Energy cost saving, Mass transfer coefficient, Rubber wood lumbers

## Abstract

Kiln drying of rubberwood lumbers is a complex transport phenomenon for realistic modeling and simulation. To decouple this complexity, researchers usually divide their research into two parts. The first one is single-lumber drying kinetics to describe how wood lumber responds to its surface conditions. Then they combine this drying kinetics with a lumped transport model or dispersion model or computational fluid dynamics. The mathematical models are then solved numerically to predict the industrial kiln drying behaviors. This work focuses on the drying kinetics of stacked rubberwood lumbers using hot air at different air velocity (0.5, 1.5, 2.5, 3.5, 4.0 m/s), relative humidity (6–67% relative humidity (RH)) and temperature (60–100 °C). The drying kinetics followed the conventional drying theory. However, the two drying periods, namely constant and falling rate (CRP and FRP), were not distinct. As the air velocity increased, the transition from CRP to FRP is faster. The middle of the transition period (at critical moisture content, CMC) moves closer to the fiber saturation point (FSP). The overall mass transfer coefficients in the falling rate period for stacked rubberwood drying were lower than those predicted by the Ananias correlation. Hence, a modified formula was proposed, representing the overall moisture transfer coefficients as a function of air velocity, temperature, relative humidity, and lumbers thickness for the range of variables under investigation satisfactorily. In general, the drying rate and the overall moisture transfer coefficient increased with increasing air velocity, drying temperature, and decreasing RH. Relative humidity directly affects the driving force of moisture transfer rate because higher RH is associated with higher equilibrium moisture content. A lumped parameter model for kiln drying was also developed. After being integrated with the estimated mass transfer coefficient, the model can predict the moisture profiles in lab-scale kiln drying satisfactory, although the model needs more validation data. These kinetic parameters and correlation for stacked rubberwood drying can be used in more complex models and process optimization in future research.

## 1 Introduction

Currently, Thailand is the largest natural rubber producer, followed by Malaysia and Indonesia in rubber plantation and production [1,2]. It is also the largest exporter of raw rubber, rubberwood, and rubberwood products. In 1979, the Thai government issued the legislation on forest closure due to deforestation, making timber harvesting from most forest illegal. The forest areas in Thailand have continued to reduce approximately 0.02% every year and stabilized to 31% of the total country area. Since then, the utilization of rubberwood for wood products has gradually expanded until the last two years. The rubber-wood industries have faced a new challenge of declined export due to the international trade-war and global economic downturn. Still, Thailand is currently the world-leading exporter of rubberwood products [3]. However, this temporal disruption is anticipated to relax soon because of the need for more renewable sources of wood from residues in long-term projection. In this critical period, efficiency and competitiveness are paramount importance for industries to survive the crises [4].

In the rubber plantation life-cycle, the farmers start taping the rubber latex from the rubber trees after 5–7 years of cultivation. As the rubber trees reach 25–30 years old, the latex yield has exhausted, and the trees become uneconomical for latex taping any longer [5]. It is then required to cut down the trees for re-plantation, leaving the residues, including leaves, branches, wood logs, and roots for proper utilization. These residues are utilized for producing wood lumbers, furniture, and for energy production. Previous research focuses on the life cycle of rubberwood [6]. Its truck (wood logs) is the most valuable raw material for dried wood lumbers and wood products, such as structural wood and furniture. In 2018, this part of rubber trees' residue alone contributed to five billion Bahts from exporting rubber-wood related products, earning from various wood products, including dried processed wood lumbers, which contributed to approximately 1.3 billion Bahts [7,8].

Presently, there is a very high competition in the export-oriented rubberwood industry. To survive, wood processing factories must operate at their best efficiency, reducing energy costs and waste, and, if possible, turns waste into value-added products. In standard rubberwood processing, the wood logs were sown, pressurized in chemical solutions, and dried in hot-air kilns. More than a hundred rubberwood lumber factories around Thailand [9] operated for more than ten years. Most of them know how to produce high-quality lumber products met with the market demand but are still struggling to reduce the total production costs to be more profitable in highly competitive markets where the buyers have increasing bargain power.

In this industry, energy cost is very high (comprises more than15% of operating expenses). Traditionally wood drying process consumes more than 60% of total energy usage, mainly in heat and electricity [10]. As the rubberwood industry is very significant for Southern Thailand and provides the primary export earnings for the local industry, there are extensive research activities carried out by Thai researchers [5,10, 11, 12, 13, 14, 15, 16, 17, 18, 19, 20, 21, 22, 23, 24]. Some of these researches focus on novel drying processes [14,16,17,19,22,25]. The others work on the effects of drying conditions on the dried products [5,11,12,14,15,26, 27, 28, 29]. A few published articles investigated moisture equilibrium relationship, drying kinetics, drying time reduction, drying process optimization, and energy efficiency in lab-scale and industrial-scale driers [10,18,21,24,30, 31, 32, 33, 34].

Srivaro et al. [5] developed an effective drying schedule for rubberwood lumber by elevating the drying temperature from 60 to 90 °C and explored the effect of pre-streaming, pre-drying, and top-loading on drying characteristics and lumber quality (30-mm thick lumber). Maintaining 30–35% RH in the drying chamber found that the drying time was shortened from 117 h to 54 h without significant quality loss, but the energy cost increased by 22%. Ratanawilai et al. [22] attempted to reduce an industrial kiln's drying time for drying rubberwood lumber (1, 1.5, and 2.0-inch thickness) by manipulating the air recycle ratio and achieved 23, 21.8, and 15.6 % time reduction respectively. However, these works did not use the kinetic modeling approach in their studies. Recently, Khamtree et al. [20] developed an empirical model for drying kinetics of rubberwood having 2.5 × 110 × 0.76 cm^3^ dimension in 80–100 °C temperature range and found that among five exponential-type models, Handerson and Pabis model gave the best fit.

However, most of the engineering researches are scattered, fragmented, and the models and parameters obtained from the published studies are not readily suitable for simulating the industrial-scale drying processes. Local rubberwood factories have no reliable tool for process design and predicting the performance of their wood drying processes affected by varying conditions of raw material, inlet air, air velocity passing the lumbers, air humidity inside the driers, etc. Hence, most of the industrial-scale kiln driers operate at conservative, sub-optimal conditions simply to be on the safe side. Consequently, a lot of energy used is wasted because of unnecessarily long drying time (up to 7 days), making it less competitive because of the high product cost.

Kiln-drying process optimization by an experimental approach, particularly on a commercial scale, is very costly and sometimes not only impractical but also too disruptive. An alternative approach is to study drying kinetics of single or uniform wood lumbers with specific physical characteristics (i.e., dimensions, orientation, macro and micro-structure of wood, density, etc.), exposed to series of the well-defined external environment (i.e., air velocity, temperature, relative humidity, etc.). The resulting drying curves are then analyzed to obtain key process parameters such as moisture sorption isotherm, specific drying rate, fiber saturation point (FSP), overall mass transfer coefficient for each drying regime, and moisture diffusivity. In turn, one can use these critical parameters in more complex mathematical models. It can incorporate kiln airflow, wood stack arrangement, inlet-air temperature-humidity profile, and hot-air recycle ratio to predict how each wood lumber responds to the imposed environment. This approach has many advantages, namely: minimal research and development cost, shorten the experiment time, generated infinite scenarios, minimum process disruption, and so on. However, the approach requires accurate parameter estimation to be realistic, employing sophisticated and distributed mathematical models. This comprehensive approach requires researchers and practitioners who are well-equipped with advanced knowledge of transport phenomena in drying processes, numerical mathematics, and related engineering software, and they are scarce. Thus, this practice is rare in drying research and industry. Some researchers chose this approach for their wood-drying modeling work with some success, however [35, 36, 37, 38, 39, 40, 41, 42, 43, 44, 45]. Some researchers suggest that empirical modeling is more accessible and practical [46].

Therefore it is desirable to find a balanced approach with a moderate mathematical requirement and does not need to provide an air velocity profile within the drying chamber. It can still describe and sufficiently predict rubberwood kiln driers' performance for different lumber properties and dimensions, drying temperature, air properties, velocity, and recycle ratio. This article is a step toward this goal. We approach the problem using a lumped-parameter model, moisture-air-lumber equilibrium, and overall moisture transfer coefficients for stacked lumbers in a kiln drier. This approach has been used successfully by some drying modeling works across different materials and heating sources [47, 48, 49, 50, 51].

One of the most significant wood drying parameters, which is difficult to predict theoretically, is the overall mass transfer coefficient (*K*) for all drying periods: initial period, constant drying rate, and falling rate period. Special attention has been emphasized in the falling rate period (after moisture content is lower than CMC). This period is usually the longest in the kiln drying process, attributed to the most considerable portion of the process energy cost. In this study, the authors chose to tackle the problem by characterizing the drying kinetics of stacked rubberwood lumbers. Special attention is on the modeling of drying rate in a constant rate period and the overall mass transfer coefficient in the falling rate period as a function of air velocity, RH, temperature, and lumber thickness. However, a few alternative approaches are available, such as those based on Weibull and Bi-Di models [52, 53, 54, 55, 56, 57]. We choose Ananias's approach because of its simplicity, flexibility, and coverage of these four process variables [58, 59, 60].

These model parameters are fundamental for more complex, realistic, practical modeling and simulation of the kiln drying process. Thus, we developed a lumped-parameter model with air recycling to mimic an actual kiln drier and illustrated how to predict industrial rubberwood drying kiln performance. Therefore, this article's main contribution is to establish a correlation for estimating the overall moisture transfer coefficients in conventional kiln drying of rubberwood lumbers as a function of drying temperatures, air velocity, relative humidity, and to a small extent, the lumber thickness. Not only this article shows that Ananias's approach needs significant modification to be suitable for rubberwood drying, but it also illustrates how to use the newly developed correlation to predict the drying kinetics in a small kiln drier. We also discuss the drying kinetics and drying mechanisms extensively to justify the applicability of the unified (covering a full range of drying curves) moisture transfer coefficient based on Ananias's approach.

## 2 Materials and methods

### 2.1 Modeling and optimization research related to current industrial practices in rubberwood drying

As mentioned earlier, Khamtree et al. [20] developed an empirical model for rubberwood drying in an 80–100 °C temperature range. They found that Handerson and Pabis model, which has the following, gave the best fit.

(1) $$\text{Moisture ratio }\left(\text{MR}\right)\;=\frac{X-X^{*}}{X_{0}-X^{*}}=A\;\exp\left(-kt\right)$$

where $X_{0},X^{*}$ and *X* are initial, equilibrium, and instant moisture content of the lumbers. *A* and *k* are the model's parameters.

This equation (Eq. (1)) conforms to the solution of diffusion theories with constant diffusivity of an isotropic solid having arbitrary shapes in batch and counter-current modes of extraction/infusion [61]. Thus the diffusion theory with appropriate boundary conditions can approximate the drying curves. The influence of the constant rate period was minimal. However, MR-vs-time curves from industrial and pilot-scale lumber kiln driers, in current practices, clearly show a significant portion of constant-rate periods [5,22]. In general, neglect the constant-rate period in the modeling of rubberwood drying.

### 2.2 Wood drying kinetics and the overall mass transfer coefficients

Ananias et al. [60] discussed the influence of various factors on the overall resistance to moisture transfer in wood lumbers and proposed a general correlation for the global mass transfer resistance (*K*).

(2) $$\frac{1}{K}\;=\alpha +\beta \;\exp\left(\frac{-z}{X_{FSP}-\;X^{*}}\right)$$

where

(3) $$\alpha \;=\;a_{0}\;\exp\left(\frac{c_{0}}{T_{K}}\right)e\text{ and }\beta \;=\;b_{0}\;\exp\left(\frac{c_{0}}{T_{K}}\right)\upsilon^{-p}$$

In Eq. (2), $X_{FSP\;}-\;X^{*}$ is the gap between the fiber saturation point and the equilibrium moisture content at specific air temperature and relative humidity. *T*_*K*_ is the air temperature in Kelvin (K) and *e* is the lumber thickness (mm). Then Eq. (2) is valid as long as *z* is not zero (that is when RH approaches 100%). However, Ananias and co-workers [60] underline that the fiber saturation point is not well-defined as the value of *X∗* at RH = 100 % is not stable.

Ananias et al. [65] determined the parameters of Eqs. (2) and (3) for spruce (*Picea abies*) and beech (*Fagus sylvatica*) during a whole kiln drying schedule (but from a single lumber) and gave the following values: *X*_*FSP*_ = 0.3 (kg moisture/kg dry wood), *p* = 0.8, *a*_*0*_ = 0.12 (m.s.kg^−1^), *b*_*0*_ = 23.9 (m.s.kg^−1^) and *c*_*0*_ = 2683 (K). After substituting these parameters into Eqs. (2) and (3) we have Eq. (4).

(4) $$\frac{1}{K}\;=\;0.12\;\exp\left(\frac{2683}{T_{K}}\right)e\;+\;23.9\;\exp\left(\frac{2683}{T_{K}}\right)\upsilon^{-0.8}\;\exp\left(\frac{\frac{RH}{100}\;-\;1}{X_{FSP\;}-\;X^{*}}\right)$$

### 2.3 Model development

In this work, the authors anticipated that Ananias's formula for specific woods in Eq. (4) developed for spruce and beech could not be applied to rubberwood drying directly, particularly when rubberwood arranges lumbers as stacks or consecutive linings. However, its generalized form in Eqs. (2) and (3) should be applicable for any type of wood if the operating conditions are not too far from conventional air drying.

### 2.4 Sorption isotherm of wood lumbers

Wood is a hygroscopic lignocellulosic material. Its moisture content will reach the equilibrium moisture content (EMC) when exposed to an environment where relative humidity (RH) is stable at a constant temperature [62]. In wood drying literature, the most widely used moisture sorption model is due to Hailwood and Horrobin. Many researchers use it as a standard model for wood drying in USDA Wood Handbook [10,63,64]. Theppaya and Prasertsan [10] covers the EMC of rubberwood in the 0–120 °C range extensively. They suggested that Haiwood and Horrobin model can represent the experimental data very well. However, they also proposed their model with comparable accuracy. Hence, in this work, we adopted a variant of the Hailwood and Horrobin model for sorption isotherm of rubberwood. The sorption isotherm has been established for wood lumber and was verified for rubberwood lumbers in which, percentage of equilibrium moisture content (EMC) expressed by Eq. (5) [10].

(5) $$X^{*}=\;EMC\left(\%\right)\;=\;\frac{1200}{W}\left[\frac{Kh}{1-Kh}+\frac{K_{1}\;Kh\;+\;2K_{1}\;K_{2}\;K^{2}\;h^{2}}{1+K_{1}\;Kh\;+\;K_{1}\;K_{2}\;K^{2}\;h^{2}}\right]$$

Where *h* is the relative humidity (decimal), and the parameters *W, K, K*_*1*_*,* and *K*_*2*_, depend on temperature (T) is in °C (Zelinka et al. [65]).

$$\begin{array}{ccccccc} W & = & 349 & + & 1.29T & + & 0.0135T^{2}\\ K & = & 0.805 & + & 0.000736T & - & 0.00000273T^{2}\\ K_{1} & = & 6.27 & - & 0.00938T & - & 0.000303T^{2}\\ K_{2} & = & 1.91 & + & 0.0407T & - & 0.000293T^{2} \end{array}$$

We will use this relation to estimate the equilibrium moisture content (*X∗*) in the following development.

### 2.5 Simple drying kinetics

Many materials, when exposed to hot air, follow simple drying kinetics [66]. This type of drying kinetics states that after a short initial period, which depends on the moisture distribution when the drying process starts, the drying rate is approximately constant. It is called *"the constant-rate period".* Theoretically, the transition from the *"constant-rate period"* to the *"falling-rate period"* was completed at the fiber saturation point (FSP). In *"the falling rate period"*, the drying rate decreases exponentially, approaching zero as the moisture content reaches equilibrium moisture content (*X∗*). Although the definition of FSP has been debated until now [66], the most fundamental definition based on solution thermodynamics was due to Zelinka et al. [65]. This article defines *"the critical moisture content"*, which is not FSP, as the best-fit moisture content at which the drying mechanism changes from CRP to FRP. We attempt to represent the whole drying curve with a single semi-empirical formula, which combined the rate equations of the constant-rate period and falling-rate period using a switching function. Previous works such as [67,68] also use related approaches, but the current model is more intuitive and fits our data very satisfactorily.

#### 2.5.1 Constant rate period (CRP)

For the constant rate period, Eq. (6) defines the drying rate.

(6) $$\Phi =-\frac{M_{0}}{A}\frac{dX}{dt}\text{ or }\frac{dX}{dt}=-\Phi \frac{A_{0}}{M_{0}}=-k_{1}$$

where *M*_*0*_, *A*_*0*_, and are lumber dry mass (kg) and its moisture transfer area (m^2^).

Since the drying rate of this period is constant, Eq. (6) follows zero-order kinetics, and Eq. (7) provides the solution.

(7) $$X=X_{0}-k_{1}t=X_{0}-\Phi \frac{A}{M_{0}}t$$

#### 2.5.2 Falling rate period (FRP)

Following the Ananias's approach/a simplified diffusion theory, we propose that a constant mass transfer coefficient is valid to describe the drying rate for the whole falling rate period, as shown in Eq. (7).

(8) $$\Phi =-\frac{M_{0}}{A}\frac{dX}{dt}=K\left(\text{X}-\text{X∗}\right)\text{ for X<}{\text{X}}_{\text{s}}$$

where $X_{s}$ is the critical moisture content which separates the CRP from FRP.

Eqs. (9) and (10) were obtained, as shown below.

(9) $$X\;=\;X*+\left(X_{0}-X*\right)\exp\left(-k_{2}t\right)\;=\;X*+\left(X_{0}-X*\right)\exp\left(-\frac{A}{M_{0}}Kt\right)$$

The overall mass transfer in Eq. (8) is equivalent to the following relations obtained from the quasi-steady-state portion of diffusion theory for infinite slab [61].

(10) $$K=q_{1}^{2}\frac{\gamma }{\gamma +1}\frac{D}{a}=\left(\frac{2.4391-0.37766/\gamma +0.021857/\gamma^{2}}{1+\left(2.293-0.4036/\gamma \right)Bi^{1.069}}\right)\frac{D}{a}$$

where $q_{1}^{2}$ is the first eigenvalues of the solution of diffusion theory.

γ is the volumetric ratio between air and the wood stack in the drying chamber

*a* is half of the lumber thickness.

Bi is the Biot number defined as $k_{m}a/D$$k_{m}$is the convective mass transfer coefficient (m s^−1^). It is a function of air velocity at the wood surface and can be estimated by an appropriate correlation [69,70].

*D* is the apparent moisture diffusion coefficient inside the wood (m^2^ s^−1^).

Note that many articles in the drying literature estimate the overall mass transfer coefficient or Biot number from the kinetic data of single lumber/solid and moisture diffusivity in the solid [3,69, 70, 71, 72, 73, 74, 75]. However, the Ananias formula covers the most critical parameters and variables, whereas most of these works either cover a limited set of variables. In this article, we prefer the direct estimation of *K* using the best-fit parameters of Eqs. (8) and (9) and develop a specific correlation through a modified version of Ananias formula.

Combining n.(7)ton.(9), after inserting a suitable (and flexible) switching function, we obtain the following drying equation covering both constant and falling rate periods.

(11) $$X\;=\;\left[\frac{1}{2}+\frac{1}{\pi }\tan^{-1}(\kappa \left(t_{s}-t\right))\right]\left(X_{0}-\;k_{1}t\right)+\left[\frac{1}{2}+\frac{1}{\pi }\tan^{-1}(\kappa \left(t-t_{s}\right))\right]\left[X*+\left(Xs\;-\;X*\right)\exp(-k_{2}\left(t-t_{s}\right))\right]$$

Where $g_{1}=\frac{1}{2}+\frac{1}{\pi }\tan^{-1}\left(\kappa \left(t_{s}-t\right)\right)$ and $g_{2}=\frac{1}{2}+\frac{1}{\pi }\tan^{-1}\left(\kappa \left(t-t_{s}\right)\right)$ are the switching functions facilitating the smooth transition from CRP to FRP, κ is the best-fit parameters of the functions, and $k_{2}=AK/M_{0}$.

$t_{s}$ is the time at which the drying mechanism is in a middle way, changing from a constant rate period to a falling rate period. Parameters in Eq. (11) were obtained by the least square fitting algorithms available in Qtiplot were used.

#### 2.5.3 Calculation of overall mass transfer coefficients: (K)

The overall mass transfer coefficients: (K) can be calculated from the basic definition [61], which includes the effect of both in-solid diffusion and surface mass convection, as shown in Eqs. (12), (13), and (14).

(12) $$K=-\frac{M_{0}}{A\left(\;X-X*\right)}\left(\frac{dX}{dt}\right)$$

or for the model

(13) $$K_{\mathrm{mod}}=\;-\frac{M_{0}}{A\left(\;X\;-\;X*\right)}{\left(\frac{dX}{dt}\right)}_{\mathrm{mod}}$$

and from experimental data

(14) $${K\;}_{\exp}=\;-\frac{M_{0}}{A\left(\;X\;-\;X*\right)}{\left(\frac{dX}{dt}\right)}_{\exp}$$

### 2.6 Lumped parameter drying model

**Figure 1:**
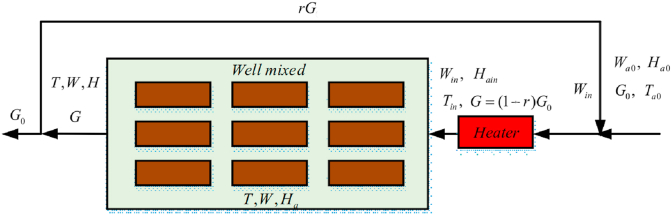
The schematic diagram of the kiln drier used for the development of the lumped parameter drying model.

This work will demonstrate how the resulting formula for predicting *K* can predict kiln driers' performance for various operating conditions. Many models have been developed for this purpose [76, 81]. Full models using CFD (computational fluid dynamics) is beyond the extent of this study. Here, we will use the so-called *"Lumped parameter model"* to illustrate the applicability of the concept of "*constant K in falling rate period*". This model states that the drying rate is linearly proportional to its driving force: drying potential, which is $\bar{X}-X^{*}$, and the overall mass transfer coefficient. The model also assumes that the mass and energy transfer occur only in one direction. The moisture content in wood at drying starting time is uniform, and the air in the drier is well-mixed (uniformly distributed). The heat losses and temperature change at different stack positions are negligible. Figure 1 depicts the schematic diagram of the drying process and the airflow.

From the moisture/water balance in drying air, we obtained Eq. (15).

(15) $$G\left(W-W_{in}\right)=KA\left(\bar{X}-X^{\text{∗}}\right)$$

Where $G,W,W_{in}$ are the mass flow rate of dry air, the moisture content in the air, and inlet air, respectively.

Eq. (16) or Eq. (17) show the moisture balance in the wood.

(16) $$-M_{\text{0}}\frac{d\bar{X}}{dt}=KA\left(\bar{X}-X^{\text{∗}}\right)$$

or

(17) $$\frac{d\bar{X}}{dt}=-\frac{KA}{M_{\text{0}}}\left(\bar{X}-X^{\text{∗}}\right)$$

The air enthalpy balance is shown in Eq. (18)

(18) $$\left[G\left(c_{pa}T+W\left(\Delta h_{\text{0}}+c_{pL}T\right)\right)-\left(c_{pa}T_{in}+W_{in}\left(\Delta h_{\text{0}}+c_{pV}T_{in}\right)\right)\right]=KA\Delta h_{V}\left(\bar{X}-X^{*}\right)-hA\left(T-T_{w}\right)$$

where.

$c_{pa,}c_{pv}$ are specific heat (Jkg^−1^K) of dry air and water vapor at drying temperature, respectively.

$\Delta h_{0},\Delta h_{v}$ are latent heat of vaporization (Jkg^−1^) at 0 °C and drying temperature, respectively.

$T,T_{in},T_{w}$ are temperatures of the air in drier, inlet air and the wood lumber, respectively.

*h* is the heat transfer coefficient (Wm^−2^K^−1^).

The enthalpy balance for the wood is shown in Eqs. (19), (20), (21), and (22).

(19) $$M_{0}\left(c_{pS}+c_{pL}\bar{X}\right)\frac{dT_{w}}{dt}=G\left[c_{pa}\left(T-T_{\text{in}}\right)-\left(c_{pL}T_{w}\right)\left(W-W_{\text{in}}\right)+c_{pv}\left(WT-W_{\text{in}}T_{\text{in}}\right)-\Delta h_{\text{0}}\left(W-W_{\text{in}}\right)\right]$$

or

(20) $$\frac{dT_{w}}{dt}=G^{\prime }\left[c_{pa}\left(T-T_{\text{in}}\right)-\left(c_{pL}T_{w}\right)\left(W-W_{\text{in}}\right)+c_{pv}\left(WT-W_{\text{in}}T_{\text{in}}\right)-\Delta h_{\text{0}}\left(W-W_{\text{in}}\right)\right]$$

and

(21) $$G^{\prime }=\frac{G}{M_{\text{0}}\left(c_{pS}+c_{pL}\bar{X}\right)}$$

(22) $$\frac{dW}{dt}=\frac{KA}{M_{a}}\left(X-X^{\text{∗}}\right)+\frac{G}{M_{a}}\left(W_{\text{in}}-W\right)$$

Where.

$c_{pS},c_{pL}$are specific heat of dry wood and water inside the wood, respectively.

$M_{a}$ is the total dry air in the kiln drier.

$W_{in}$ and W are the humidity of inlet air and the air inside the drying chamber, respectively.

The moisture balance in the wood is shown in Eq. (23).

(23) $$\frac{dX}{dt}=-\frac{KA}{M_{\text{0}}}\left(X-X^{\text{∗}}\right)$$

The air enthalpy balance is shown in Eq. (24) and the enthalpy balance for the wood is shown in Eq. (25).

(24) $$\frac{dT}{dt}=\frac{1}{M_{a}\left(c_{pa}+Wc_{pv}\right)}\left[G\left(H_{ain}-H_{a}\right)+M_{a}\Delta h_{0}\frac{dW}{d}-hA\left(T-T_{W}\right)\right]$$

(25) $$\frac{dT_{w}}{dt}=\frac{1}{M_{\text{0}}\left(c_{pS}+c_{pL}X\right)}\left[hA\left(T-T_{w}\right)-M_{\text{0}}\Delta h_{v}\frac{dX}{dt}\right]$$

Where $H_{a},H_{ain}$ are enthalpy of air (Jkg^−1^) inside the kiln drier and inlet air, respectively.

### 2.7 Experimental method

#### 2.7.1 Experimental set-up to study the effect of air velocity on lumbers drying kinetics

##### 2.7.1.1 Wood sample preparation

**Figure 2:**
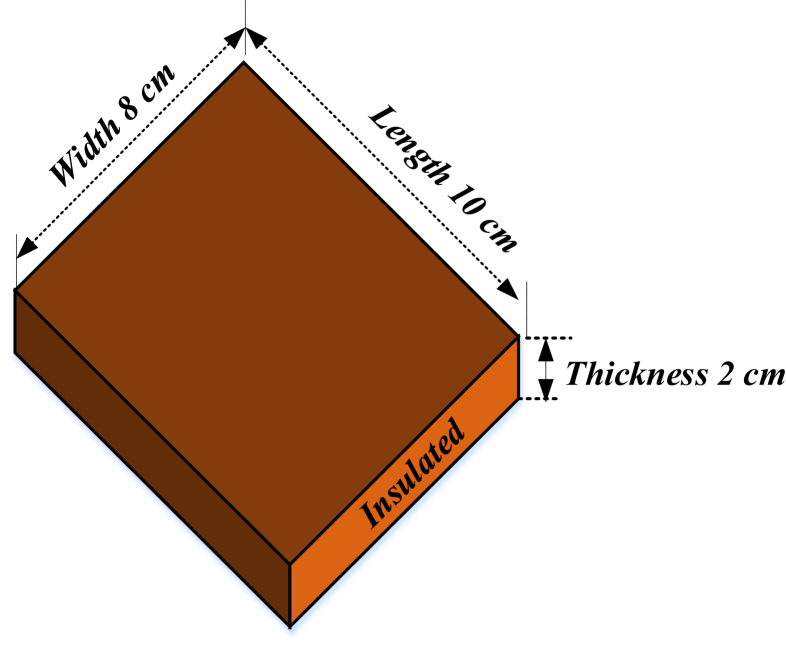
Rubberwood dimension and the direction of airflow.

**Figure 3:**
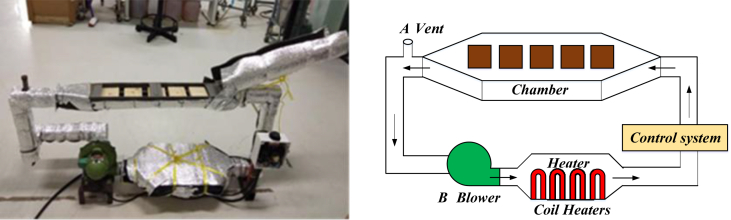
Construction wood dryer.

In these experiments, the rubberwood lumbers were prepared by sawing rubberwood logs into lumbers with 10 cm length × 8 cm width × 2 cm thickness (Figure 2). The logs were obtained from a wood processing plant. In one layer, there are five wood lumbers arranged consecutively (Figure 3). Before drying, lumbers were impregnated with boron solution. Each lumber piece was covered with wax on both longitudinal ends. The moisture can only transfer from the wood surface in perpendicular to airflow direction and airflow direction.

##### 2.7.1.2 Wood dryer: sequential arrangement mimicking one layer of rubberwood lumbers in a stack

The volume of the drying chamber was 0.05 × 0.12 × 0.60 m^3^. An electric heater and air blower were used to heat and circulate the air (Figure 3). The recirculation rate was controlled by adjusting the air outlets located at the end of the drying chamber (A) and the blower (B). Five wood lumber (8 cm × 10 cm x 2 cm) were placed into the drying chamber serially. Each lumber was separated by 2 cm space. The drier was insulated by 0.5 cm of PE sheet wrapped by an aluminum sheet.

##### 2.7.1.3 Drying conditions used in the experiment

Drying processes operate according to the experimental conditions summarized in Table 1. The temperature and relative humidity were measured using a digital-wired thermometer at 4 h intervals. The air velocity was varied in the range of 0.5–3.5 m/s at a constant temperature (70±1 °C). RH was controlled at 25% and 30%, and the wood's initial moisture content of the wood was 89.6 % (dry basic). The air velocity was measured using anemometer KIMO LV-50. Drying stopped when the wood reached less than 8–12% moisture content (dry basic).

**Table 1:** Experimental set-ups for studying the effect of air velocity in kiln drying.

Conditions	Factors		RH	EMC	Drying time
	Air velocity	Temperature	(%)	(%)	(hr.)
V1	0.5 m/s	70 °C	30	4.6	40
V2	1.5 m/s	70 °C	30	4.6	36
V3	2.5 m/s	70 °C	25	3.5	32
V4	3.5 m/s	70 °C	25	3.5	32

#### 2.7.2 The set-up of kiln drying of wood lumbers for studying the effects of temperature and relative humidity

**Figure 4:**
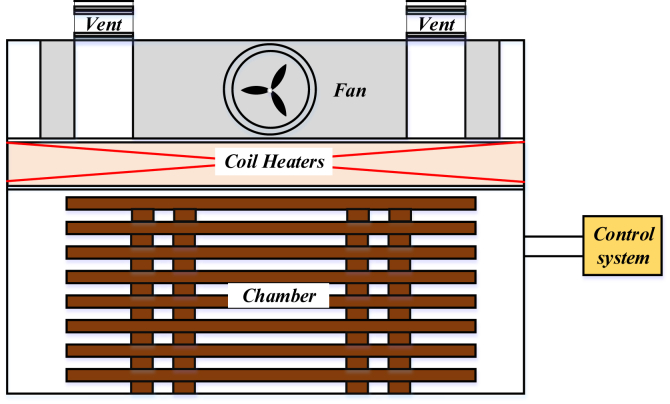
The design of wood drier and stack arrangement for kiln drying.

The authors obtained these sets of experiments and their results from Tomad et al. [11]. Figure 4 shows the construction of the wood dryer.

In these experiments, the lumbers have a dimension of 1 m length × 8 cm width × 3 cm thickness loaded in the drying chamber having a volume of approximately 1 m^3^. The stack was filled with ten layers of wood lumbers. Ten lumbers are placed parallel to each layer/s airflow direction, where the hot air is in contact with the full lumber length. The velocity of air approaching the wood stack was approximately 4.0 m/s. The drying kinetics of rubberwood in a kiln drier was studied in two sets of experiments. The first set was designed to investigate the effect of drying temperature in the 60–100 °C range (T1-T5) while the equilibrium moisture content (EMC) of wood lumbers was controlled at 3.3%. The second experimental set was designed to study the effect of relative humidity at a constant temperature (90 °C) by varying wet bulb temperature such that RH was in the range of 6–67% RH (H1–H5). The temperature and relative humidity were measured using the dry bulb and wet bulb thermometers. The experimental design for obtaining these two datasets is shown in Table 2 [11].

**Table 2:** Experimental set-ups for studying the effect of temperature and relative humidity.

Conditions	Dry-bulb (◦C)	Wet-bulb (◦C)	RH (%)	EMC (%)
T1	60	34	12	3.3
T2	70	42	18	3.3
T3	80	51	24	3.3
T4	90	60	26	3.3
T5	100	71	29	3.3
H1	90	40	6	1.0
H2	90	50	14	2.0
H3	90	60	26	3.3
H4	90	70	43	4.9
H5	90	80	67	8.1

## 3 Results and discussions

### 3.1 The effect of air velocity on lumbers drying kinetics

**Figure 5:**
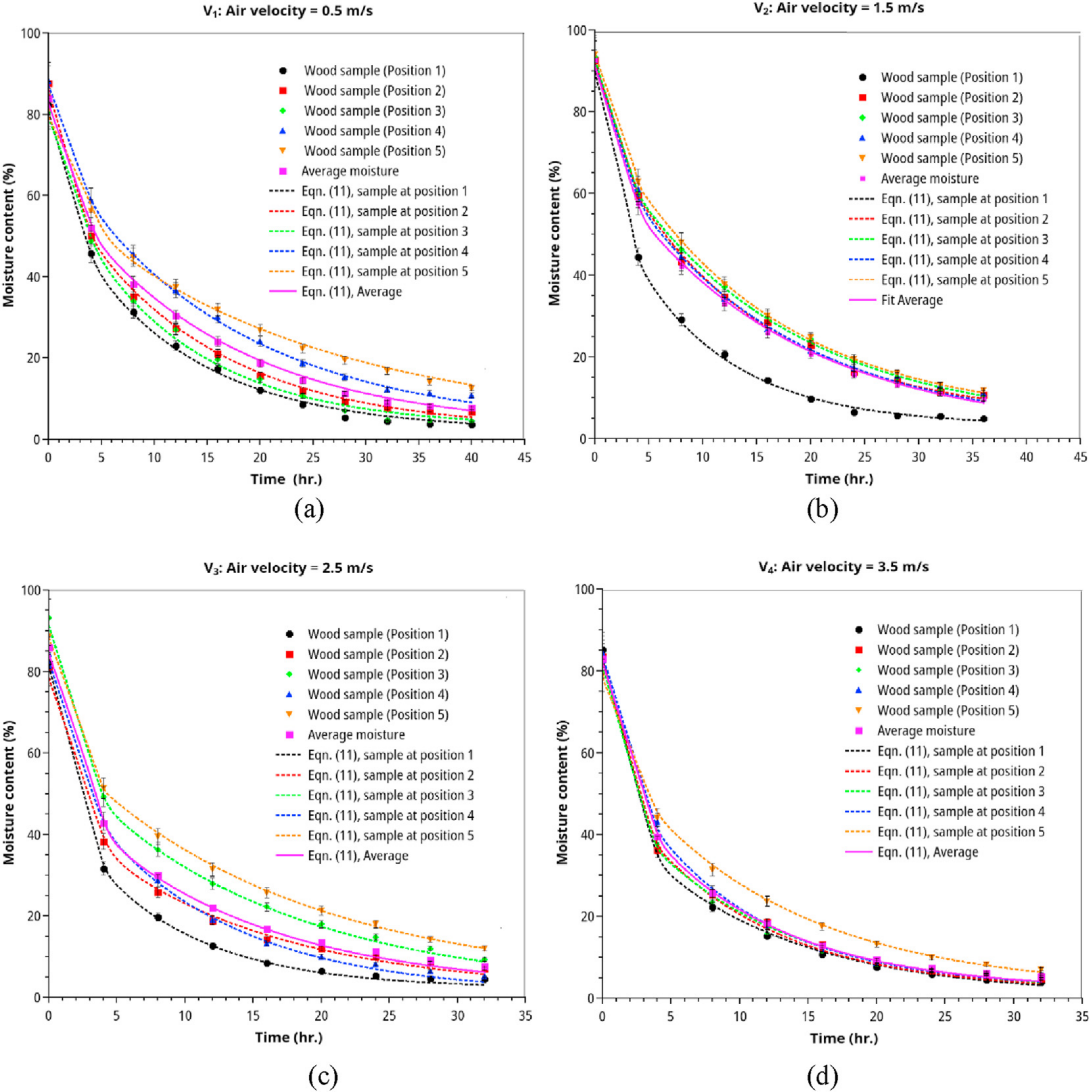
Drying curves of rubberwood lumber at five different position which are arranged sequentially along the airflow path. (a) for velocity of 0.5 m/s, (b) for velocity of 1.5 m/s, (c) for velocity of 2.5 m/s, (d) for velocity of 3.5 m/s.

**Figure 6:**
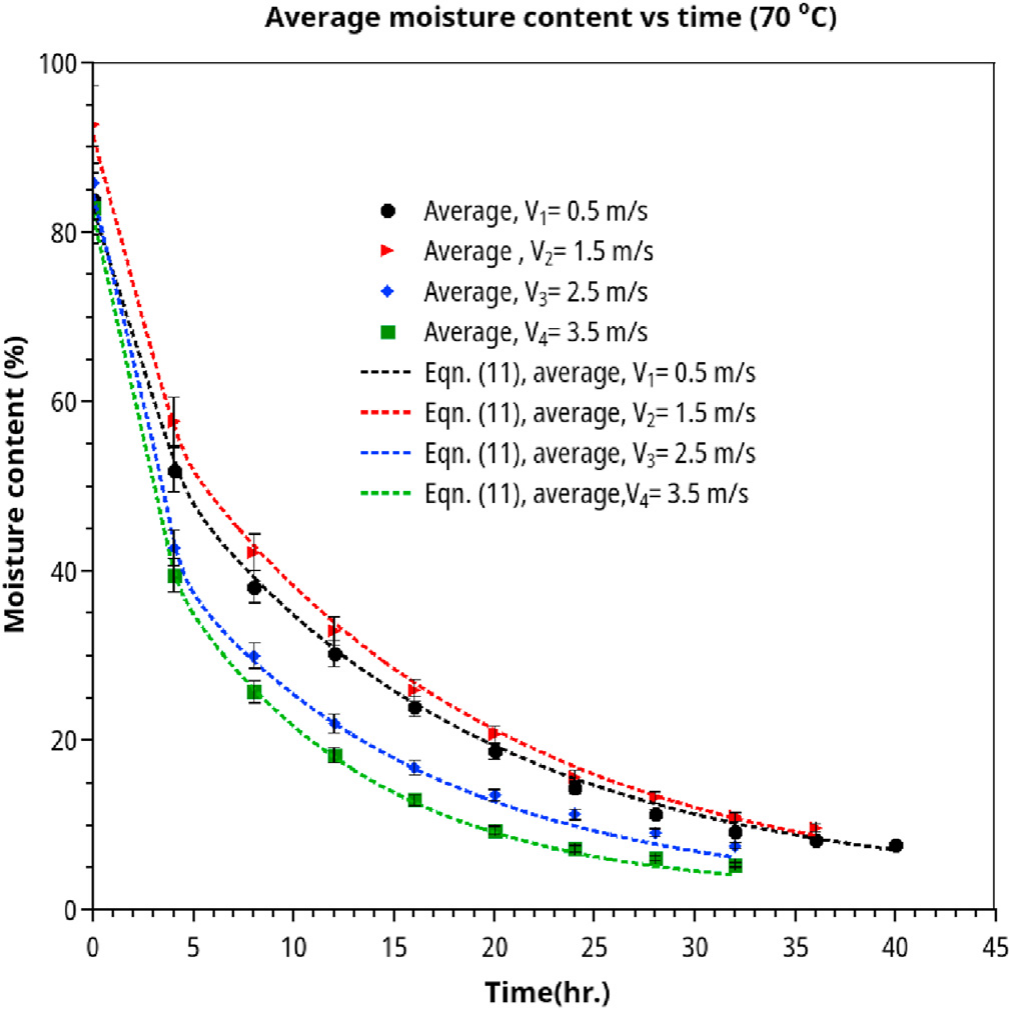
Average drying curves of rubberwood lumber at four different velocities (0.5–3.5 m/s) at drying temperature of 70 °C.

**Figure 7:**
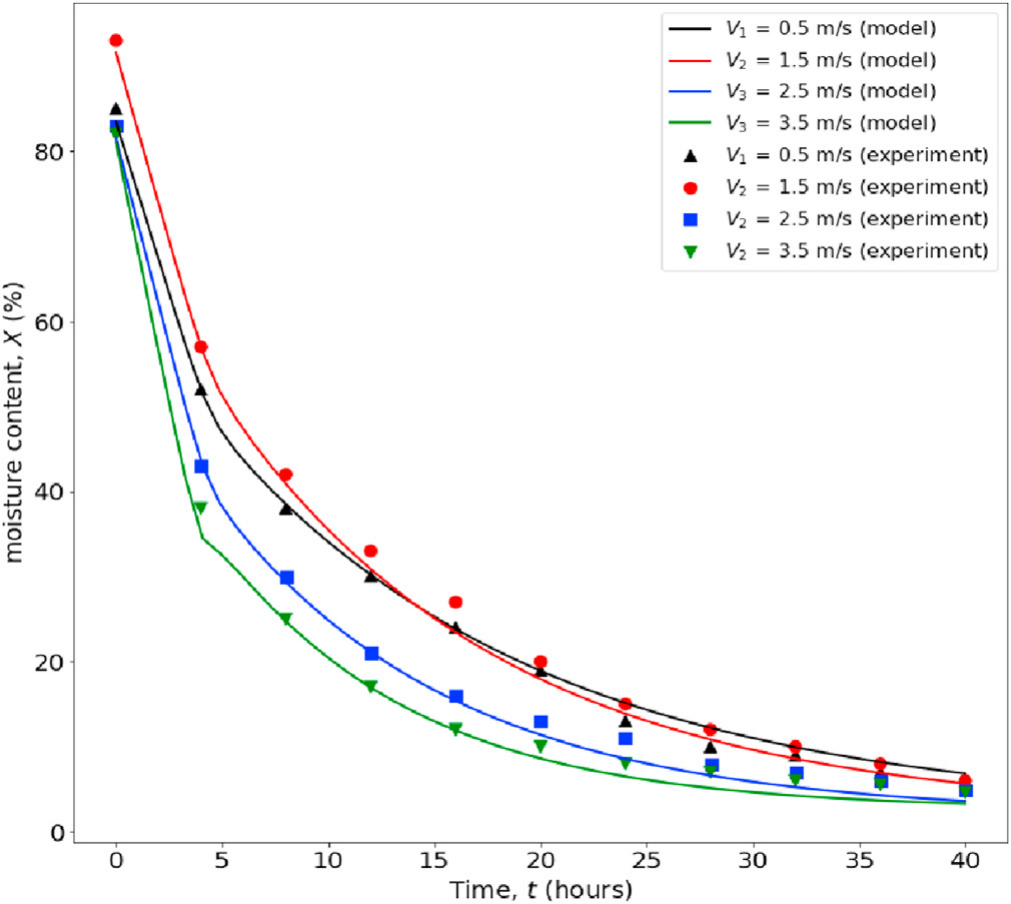
Drying curves of wood lumber in the velocities range of 0.5–3.5 m/s, drying temperature of 70 °C.

Figures 4 and 5 show the rubberwood lumbers' drying curves at four velocities (0.5, 1.5, 2.5, 3.5 m/s) and five different sequential positions along the airflow path. The combined drying curve model (Eq. (10)) fitted the experimental data very well for all drying conditions. The first position showed the highest drying rate at a specific velocity and vice versa. At low velocity (0.5 m/s, RH 30%), the position strongly affected the drying rate (Figure 5a). As the velocity went up to 1.5 m/s (RH 30%), the drying rate was less effected by position except for the first position. Similar results were observed for medium (2.5 m/s, RH 25 %) and high velocities (3.5 m/s, RH 25%). When the average moisture content was used (average of lumbers at five positions at each level of air velocity) in model fitting, we obtained the drying curves in Figures 6 and 7. The effect of velocity on drying rate was strong at low velocities (0.5–1.5 m/s) but less pronounced at higher velocities (2.5–3.5 m/s).

Table 3 summarizes the experimental data for all levels of air velocity in terms of drying rate in the constant rate period (*k*_*1*_), falling period (*k*_*2*_), and the overall mass transfer coefficient (*K*). the goodness of fit (R^2^) was excellent (R^2^ > 0.99) for all cases.

**Table 3:** The parameter estimates from the wood drying kinetics model.

Conditions	*X*_*s*_ (%)	*t*_*s*_ (hr.)	κ	RH (%)	*k*_*1*_ (h^−1^)	*k*_*2*_ (h^−1^)	*K* (kg/m^2^ s)	R^2^
V1 (0.5 m/s)	50	4.5	1	30	7.45	0.0425	6.14E-05	0.998
V2 (1.5 m/s)	62	4.2	1	30	8.76	0.0600	8.00E-05	0.997
V3 (2.5 m/s)	51	4.0	1.2	25	9.67	0.0670	8.93E-05	0.997
V4 (3.5 m/s)	45.3	3.9	2	25	10.36	0.0820	1.09E-04	0.996

### 3.2 The estimated critical moisture content (CMC) in relation to fiber saturation point (FSP)

In 1944 Tiemann defined *"fiber saturation point"* as the moisture content at which wood substance becomes saturated, and the differential heat of sorption becomes zero. To simplify the definition. Later, FSP is referred to as the stage of wetting or drying of wood at which the cell walls are saturated with bound water, and the cell cavities contain no free water [66]. A more fundamental definition based on solution thermodynamics is due to Zelinka et al. [65]. FSP of rubberwood at room 35 °C was determined according to the definition as reported by Matan et al. [13] as 26.8%. Furthermore, in the study of sorption isotherm of rubberwood in the temperature range of 0–120 °C, Theppaya et al. [10] recommended a formula to estimate the FSP as follows.

(26) $$X_{FSP}\left(\%\right)=25.7249-0.113798T+0.0017957T^{2}-1.13697\times 10^{-5}T^{3}$$

At 70 °C the calculated $X_{FSP}$ is 22.6%, much less than the critical moisture content (X_s_≈ 40%). It explains that the drying mechanism changed from evaporation-controlled (CRP) to diffusion-controlled gradually, passing through the critical moisture content (CMC) and became fully diffusion-controlled after passing through FSP.

### 3.3 The effect of air velocity on drying kinetics

In the constant drying rate period (CRP) (MC > *X*_*s*_), at a fixed drying temperature (70 °C), as air velocity increased from 0.5 m/s to 3.5 m/s, the drying rate (*k*_*1*_) got higher from 7.45 h^−1^ to 10.36 h^−1^. However, as the air velocity was higher than 1.5 m/s, the velocity has a minor effect on the increase of drying rate as the heat transfer rate to the lumber surface is balanced with the enthalpy change needed for water evaporation. Further increase in velocity will not significantly affect the drying rate because of the diminished heat and mass transfer rate due to surface temperature approaches the wet-bulb temperature, and the air becomes saturated with water vapor. Similarly, in the transition and the falling rate period (after the MC approached and passed through CMC), increasing air velocity from 0.5 m/s to 3.5 m/s also resulted in a higher drying rate (*k*_*2*_). Contrary to CRP, the effect of velocity was strong; even the velocity was as high as 3.5 m/s. The trend can be explained as the lumber stacking effect that obstructs the airflow path and reduces the turbulence at the air-lumber interface and the reduced driving force due to air humidity increase along the flow path. This effect required much stronger air turbulence to diminish. The mass transfer coefficient followed a similar trend as that of the drying rate. It is interesting to notice that high air velocity was associated with lower critical moisture content (CMC), pushing CMC toward FSP, as shown in Table 3. Consequently, air velocity plays a more significant role in the drying of stacked lumbers than single lumber.

### 3.4 The overall mass transfer coefficient (*K*) after CMC: comparison between experimental data, calculated from Ananias model [64] and the formula developed in this work

Eq. (27) represents the overall mass transfer coefficient (*K*), obtained from a partial least-square fitting of the average moisture content versus time for our available data. It is based on the general form (Eqns. (2) and (3)) proposed by Ananias et al. [60].

(27) $$\frac{1}{K}\;=\;\alpha \;+\;\beta$$

where $\alpha \;=\;a_{1}+\;a_{0}\;\exp\left(\frac{c_{0}}{T_{K}}\right)e^{m}$ and. $\beta \;=\;\left(b_{0}{\left(\frac{v}{4}\right)}^{-av^{b}}-b_{1}\right)\exp\left(\frac{c_{0}}{T_{K}}+\frac{\frac{RH}{100}-1}{X_{FSP}-X^{*}}\right)$

The best estimates of the correlation parameters were: $a_{1}=2,500$ m^2^ s,${\text{a}}_{\text{0}}=\;0.064\;{\text{kg}}^{-1}$$c_{0}=2\text{,}675\text{ K}$, m=1.23 ,a=0.492, b=0.35, $b_{0}=176.0$, $b_{1}=100.0$

Two modifications of the Ananias correlation were added to accommodate all compiled data. Firstly, the empirical parameters a_1_ and m were included in the internal resistance to mass transfer (α) to rectify the lumber thickness's non-linear effect. Secondly, the velocity term was modified empirically by adding parameters b_1_, a, and b. In the Ananias formula, the exponent of *v* (*p*) is 0.8, whereas the best-fit exponent of the available rubberwood drying (this work) was in the range of 0.386–0.8. This range covers the exponent of velocity (0.59) in a popular correlation for the convective mass transfer coefficient of moisture, as referred to in Akpinar and Dincer [52], which takes the following form (Eq. (28)).

(28) $$Bi=22.55Re^{-0.59}\;or\;k=22.55\left(D/\nu \right)V^{-0.59}$$

Where *Re* is Reynold number and *k* is the convection mass transfer coefficient. The difference in this exponent may result from the combined effect of convection, in-solid diffusion, and lumber stacking in the transition and falling rate periods.

**Figure 8:**
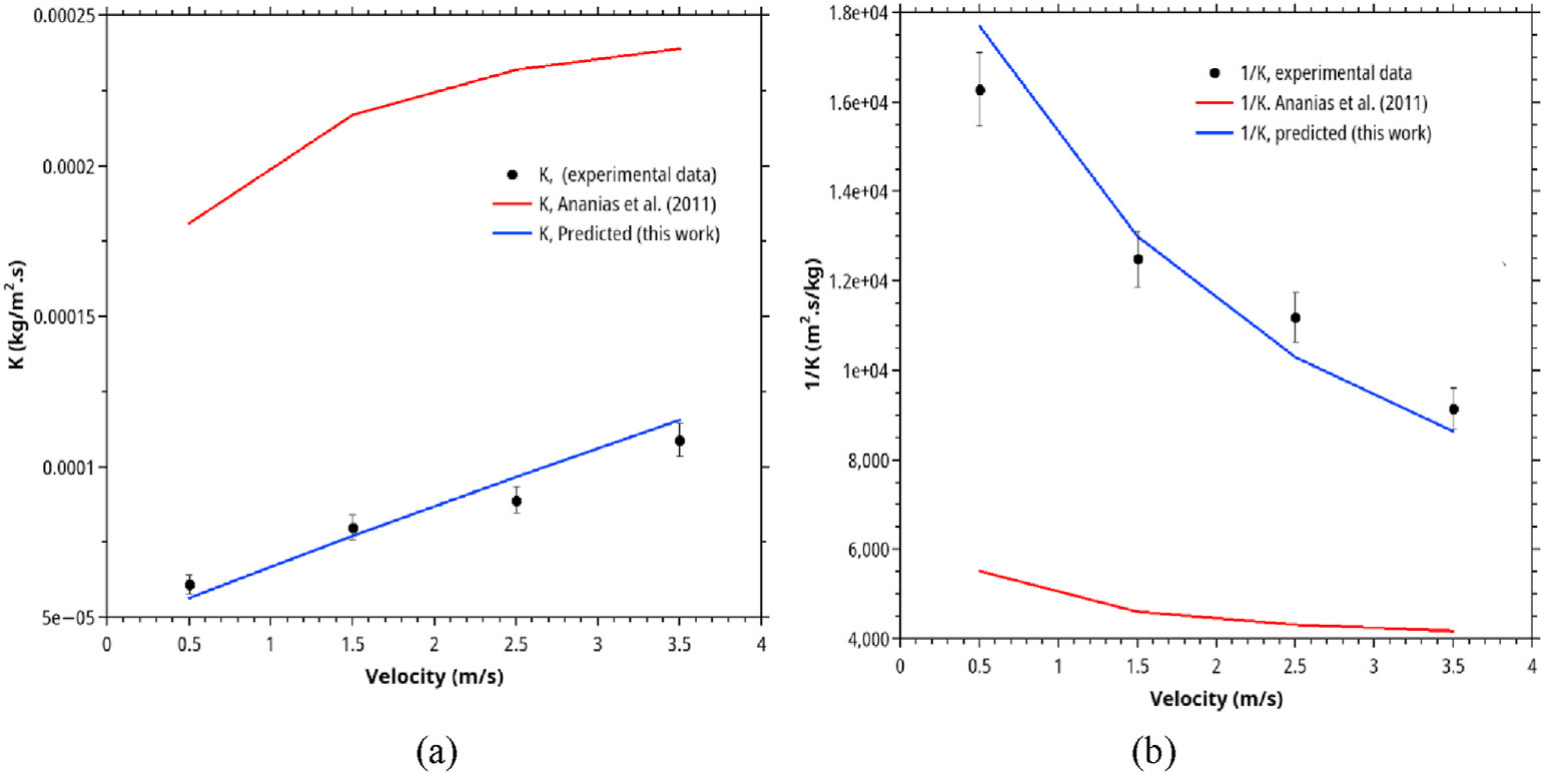
Comparison between the overall mass transfer coefficient (*K*) calculated from experimental data, Ananias correlation, and the correlation developed in this work.

To evaluate the applicability of the Ananias equation (Eq. (4)) and our proposed new formula [60] for estimating *K* in rubberwood drying, we compared the *K* from experimental data to that estimated by Eqs. (4) and (27). Tables 4 and 5, and Figures 7 and 8 show the comparative.

**Table 4:** The effect of air velocity on the overall mass transfer coefficient compares the experimental data, Ananias correlation, and the proposed correlation (Eq. (27)).

Wood Species	*e* (mm)	*V* (m/s)	Temperature (◦C)	*K* experimental (kg/m^2^.s)	*K* model predicted (kg/m^2^.s)	*K* Ananias et al. (2011) (kg/m^2^.s)
Rubberwood	10	0.5	70	6.14E-05	5.65E-05	1.81E-04
		1.5	70	8.00E-05	7.71E-05	2.17E-04
		2.5	70	8.93E-05	9.67E-05	2.32E-04
		3.5	70	1.09E-04	1.15E-04	2.39E-04

**Table 5:** Process variables and parameters of the second and third sets of experimental data intended to study the temperature and RH's effect on the drying performance. The original data were obtained from Tomad et al. [11].

Wood Species	*e* mm	*V* m/s	*T* (dry-bulb) (◦C)	*T*_*w*_ (wet-bulb) (◦C)	RH (%)	*K* experimental Tomat (2011) (kg/m^2^.s)	*K* model predicted (kg/m^2^.s)	*K* Ananias et al. (2011) (kg/m^2^.s)
rubberwood	15	4	60	34	12	9.64E-05	9.58E-05	1.66E-04
			70	42	18	1.08E-04	1.05E-04	2.05E-04
			80	51	24	1.13E-04	1.13E-04	2.49E-04
			90	60	26	1.27E-04	1.26E-04	3.03E-04
			100	71	29	1.42E-04	1.37E-04	3.60E-04
			90	40	6	1.47E-04	1.45E-04	3.18E-04
			90	50	14	1.40E-04	1.39E-04	3.14E-04
			90	60	26	1.26E-04	1.26E-04	3.03E-04
			90	70	43	9.79E-05	9.87E-05	2.76E-04
			90	80	67	5.69E-05	5.66E-05	2.11E-04

Figure 8 depicts the comparison between Ananias formula (Eq. (4)), our modified version (Eq. (27)) with experimental *K* data.

**Figure 9:**
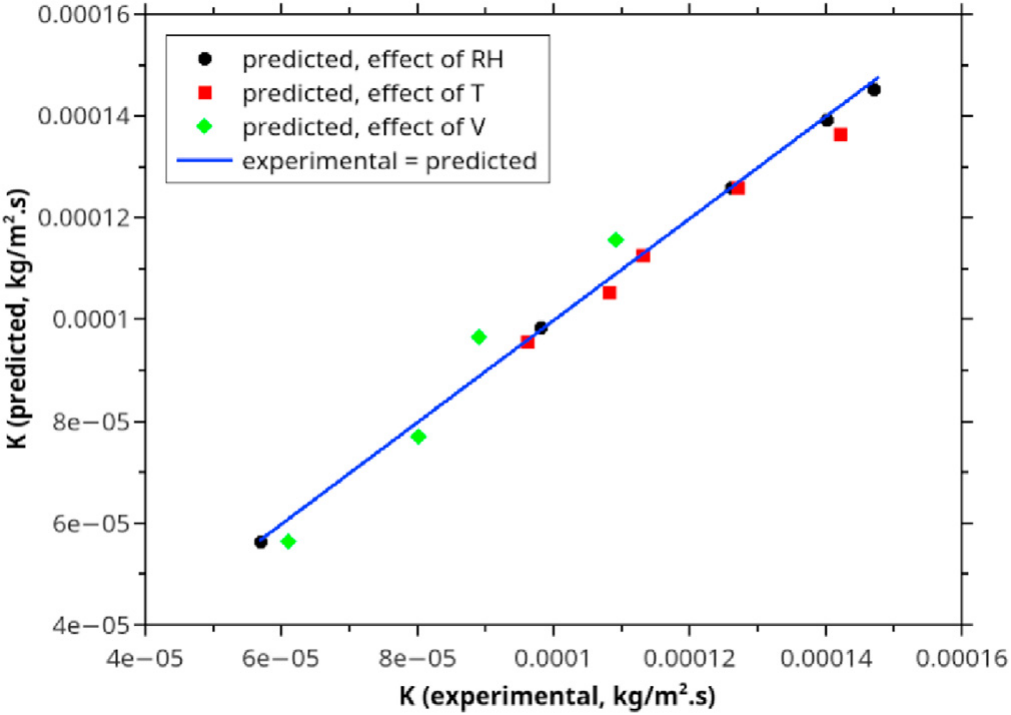
Comparison between mass transfer coefficient (*K*) obtained from three sets of experimental data and those predicted by the proposed correlation for rubberwood lumber drying.

From Table 4 and Figure 8, it is evident that the Ananias correlation is not suitable for hot air drying of rubberwood, particularly for stacked rubberwood drying because it tended to overestimate the overall mass transfer coefficient. These can be partially explained by the different rubberwood structure, which is less porous than spruce and beechwood, where Ananias derived his correlation. Furthermore, our experimental data were based on stacked or serially placed wood lumbers, not based on individual lumber. Indeed, other factors, including flow arrangement, play essential roles but can not be verified in this workresults (see Figure 9).

### 3.5 Effect of temperature and relative humidity on kiln drying

Table 5 summarizes the effects of drying temperature and RH on the overall mass transfer coefficients. Keeping the EMC at a constant value (3.3%) by adjusting RH at specific temperatures and varying the temperature in the range of 60–100 °C, as higher drying temperature was higher, the overall mass transfer coefficients increased. Conversely, at a fixed temperature (90 °C) and varying the RH from 6-67% (EMC 1–8.1%), as shown in Table 2, the overall mass transfer coefficient decreased with increasing RH. The decrease results from higher EMC at higher RH and humidity-dependence of the convective mass transfer coefficient (k). Again, Ananias correlation always over-predicted the overall mass transfer coefficients for the reasons mentioned previously.

### 3.6 Model analysis and predictions

Ananias formula base the derivation on a fundamental theory of mass transfer, which states that:

Total resistance to mass transfer = in-solid mass transfer resistance + convective resistance

or

(29) $$\frac{1}{K}=\frac{1}{K_{S}}+\frac{1}{mK_{G}}$$

where *K*_*S*_ is an in-solid mass transfer coefficient based on diffusion theory, *K*_*G*_ is the convective/film mass transfer coefficient, and *m* is an equilibrium constant. In principle, the convective resistance decreases by increasing air velocity because of the thinner stagnant films at the air-lumber interface. In contrast, velocity has an insignificant effect on the resistance of in-solid diffusion.

To compare our model prediction with a set of kiln drying data from Tomad et al. [11], we carried out the following analysis. We focus on the effects of lumber thickness, drying temperature, and relative humidity on the predicted overall mass transfer coefficients and the ratio between in-solid resistance to moisture transfer to the convective one: (1/*K*_*s*_)/(1/*mK*_*G*_).

**Figure 10:**
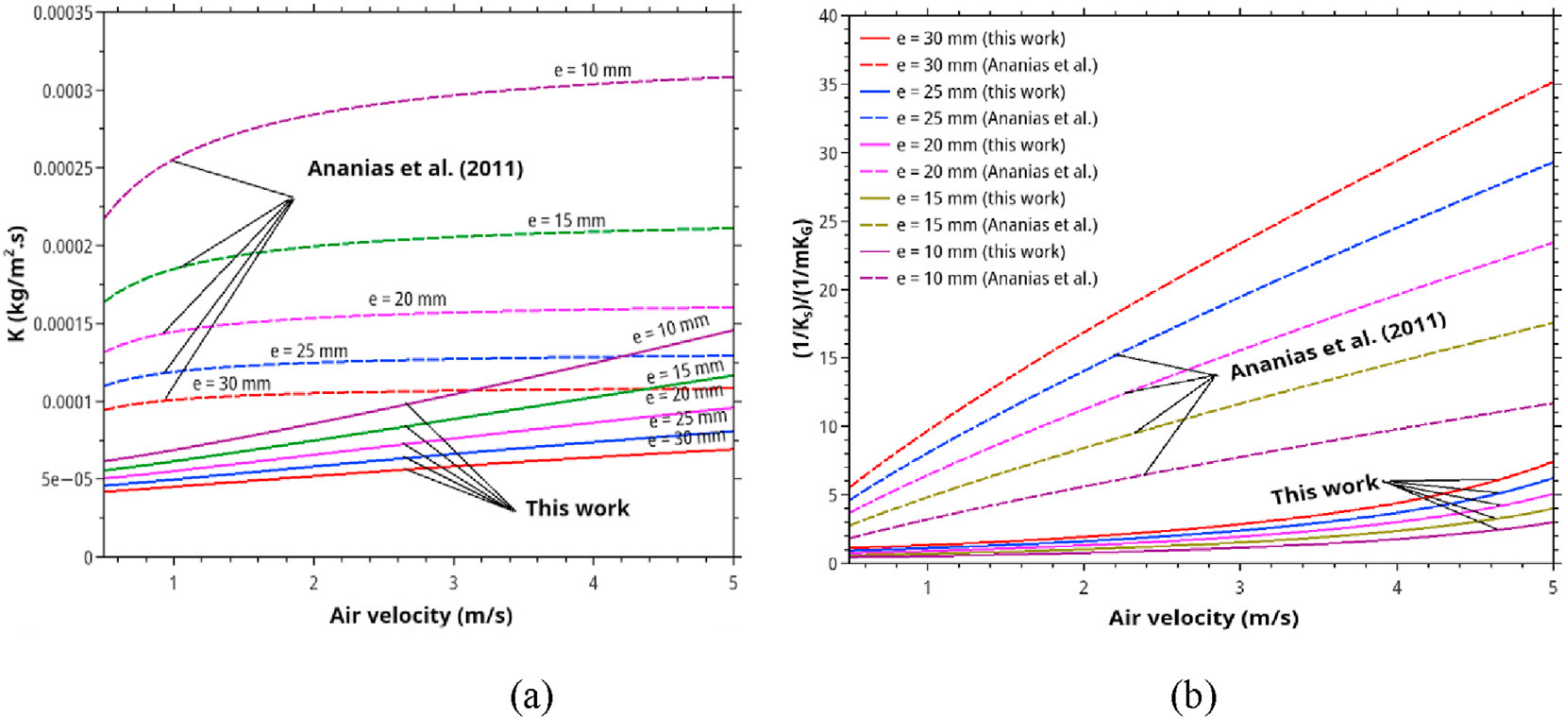
The effect of thickness and air velocity on (a) the overall mass transfer coefficients and (b) the relative importance of in-solid/convective resistances as predicted by Ananias correlation and this work. The calculation is based on *T* = 70 °C, *RH* 25% and *X∗* = 0.046.

The effect of thickness is shown in Figure 10. Thickness has a strong influence on the internal mass transfer resistance. By referring to diffusion theory, the parameter *m* should be close to unity. However, in our formula, *m* was estimated from the experimental data as 1.23. It is higher than that predicted by diffusion theory. Presumably, the actual diffusion occurred in two-dimensional rather than the assumed one dimension with only thickness as a characteristic length. The lumber dimensions used in our experiments (8 cm × 10 cm x 2 cm for the study of velocity effect and 1 m length × 8 cm width × 3 cm for the rest) was not a strictly infinite slab. Based on this value of *m*, the model predicts that increasing the wood thickness from 20 mm to 30 mm (50 % increase in thickness) will raise the internal resistance by 65% approximately, which is very pronounced. Among the variables investigated in this work, wood thickness has the most significant influence on the overall mass transfer coefficient and the drying time. This prediction has a similar trend as predicted by the Ananias formula. However, the magnitudes are slightly different because Ananias formula predicts a linear increase in the resistance with increasing thickness according to the diffusion theory.

One striking difference between *K* predicted by Ananias formula, and our correlation is the more persistent effect of velocity on *K* in our cases. This result can be explained by the stacking effect in stacked lumbers (our cases) but does not include in the Ananias formula. It implies that, for single wood lumber, the velocity of >3 m/s is enough to reduce convection resistance to an insignificant level. In stacked lumbers require much larger velocity to ensure that all lumbers expose to a near-identical drying environment. Velocity larger than 4 m/s would be impractical because of large energy requirements, however.

**Figure 11:**
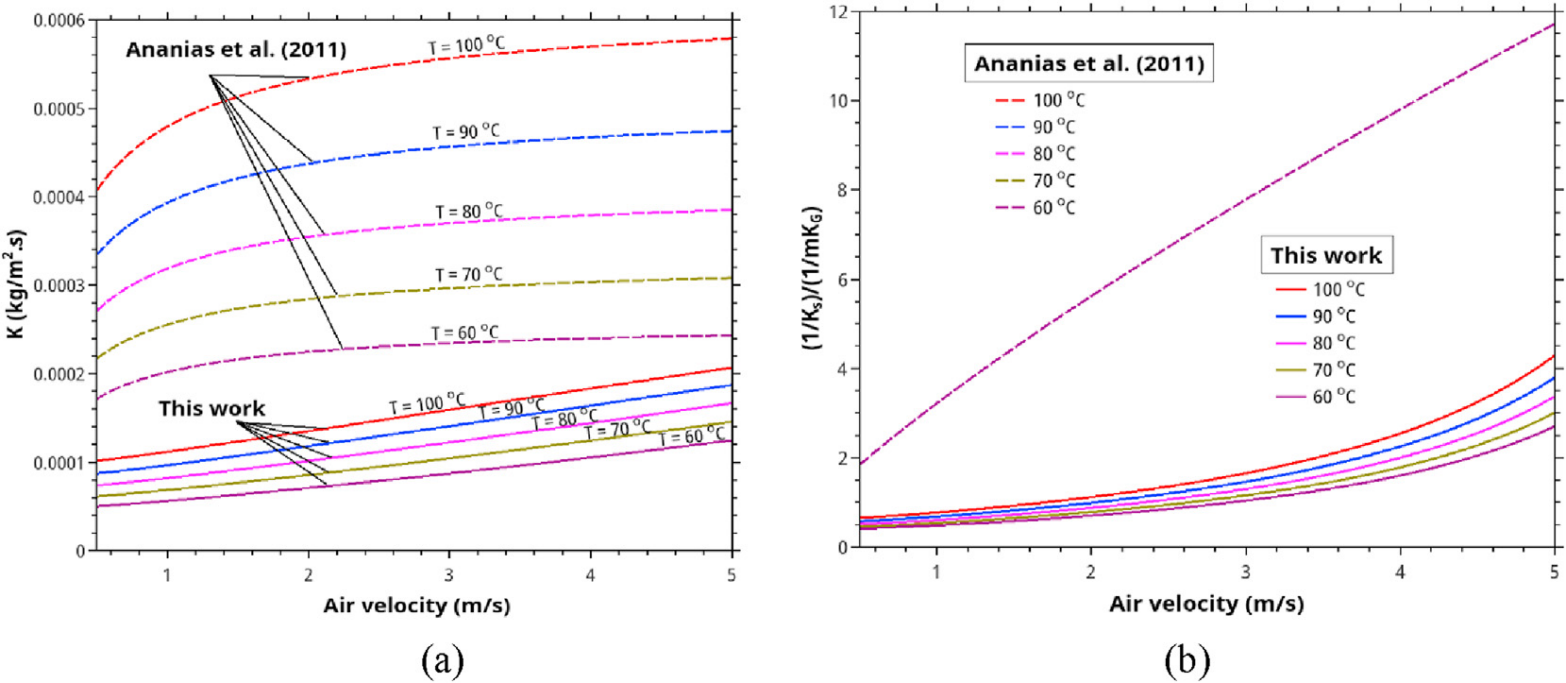
The effect of temperature and air velocity on (a) the overall mass transfer coefficients and (b) the relative importance of in-solid/convective resistances as predicted by Ananias correlation and this work. The calculation is based on *e* = 10 mm, *RH* 25% and *X∗* = 0.046.

Regarding the effect of drying temperature, Ananias formula predicted that the relative importance of internal resistance to the external one is almost temperature independent. However, as shown in Figure 11, our result for stacked lumbers, the relative importance is temperature-dependent. Both correlations predicted that increasing the drying temperature from 60 to 100 °C will approximately double the overall mass transfer coefficients. Besides, for stacked lumber, the relative effect of in-solid resistance is more pronounced at the higher velocity (i.e., *V* > 3 m/s).

**Figure 12:**
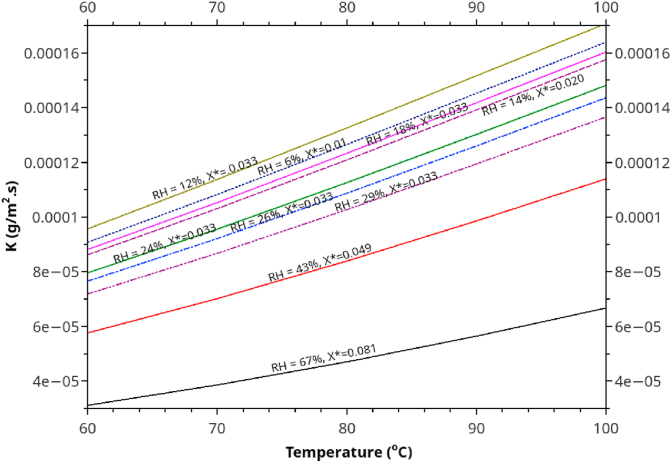
The combined effect of temperature and %RH on the overall mass transfer coefficient as calculated using the correlation developed in this work. The calculation is based on *e* = 10 mm and *V* = 3.5 m/s.

Figure 12 shows the effect of combined temperature and %RH on *K* at a fixed air velocity (4.0 m/s). In general, higher RH is associated with lower *K*. For example, at 90 °C, increasing RH from 6% to 67% reduces K from 0.00015 to 0.00005 kg m^−2^s^−1^ (two-third reduction). Regarding the effect of the %RH on K, Ananias formula and our modified version predict similar trends. We need not modify the %RH term in his original form to conform with the experimental data. Increasing %RH modifies (lowering) the driving force for moisture transfer by convection and rises the EMC at the air-lumber interface, thus indirectly limit the driving force for in-solid moisture diffusion. As %RH increases, external resistance (convection mass transfer) becomes more pronounced than the resistance due to internal diffusion. The moisture in solid particles becomes more uniform, independent of air velocity.

RH is one of the important manipulated variables in wood drying and drying of porous solid in general. It is generally desirable to keep RH at a high level at the initial period of drying to prevent cast hardening, cracks, and deformation, which will result in low quality of dried products. In terms of energy and exergy efficiency, manipulation of RH in the CRP and the transition periods is a practical measure. The recent work recommended a multistage relative humidity control strategy to be optimal for convective drying energy usage. With RH's increase in the initial drying period from 4% to 40%, the energy loss caused by exhaust air and process irreversibility could be reduced up to >50% without a significant increase in drying time.

**Figure 13:**
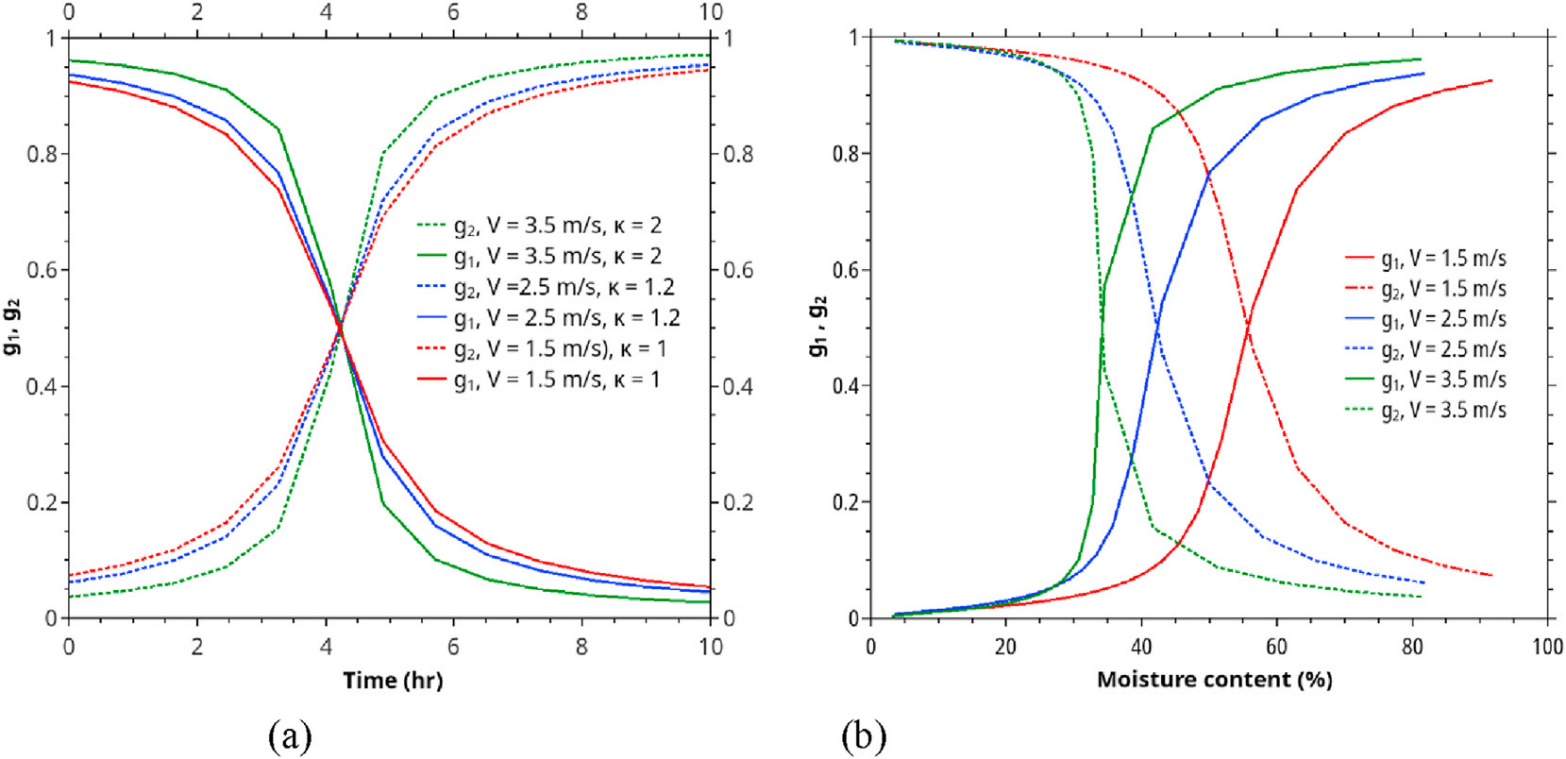
The transitional characteristics of rubberwood drying as virtualized by κ, g_1_, and g_2_.

Starting from high %RH (i.e., 40%), two-step or drop-down RH control is suitable for the products in which CRP and FRP are distinct, whereas three-steps RH or gradually RH reduction is more suitable for the products having non-distinct CRP-FRP curves. Figure 13 visualizes the transitional characteristics of rubberwood drying. The changes g_1_ (1–0) and g_2_ (0–1, or from fully CRP to FRP) occurred when the moisture changed from 0.6 to 0.3. The period took approximately 10 h. Hence, the transition is a gradual one, and an incremental RH reduction strategy would be more suitable for rubberwood drying.

### 3.7 Lumped parameter drying model prediction

**Figure 14:**
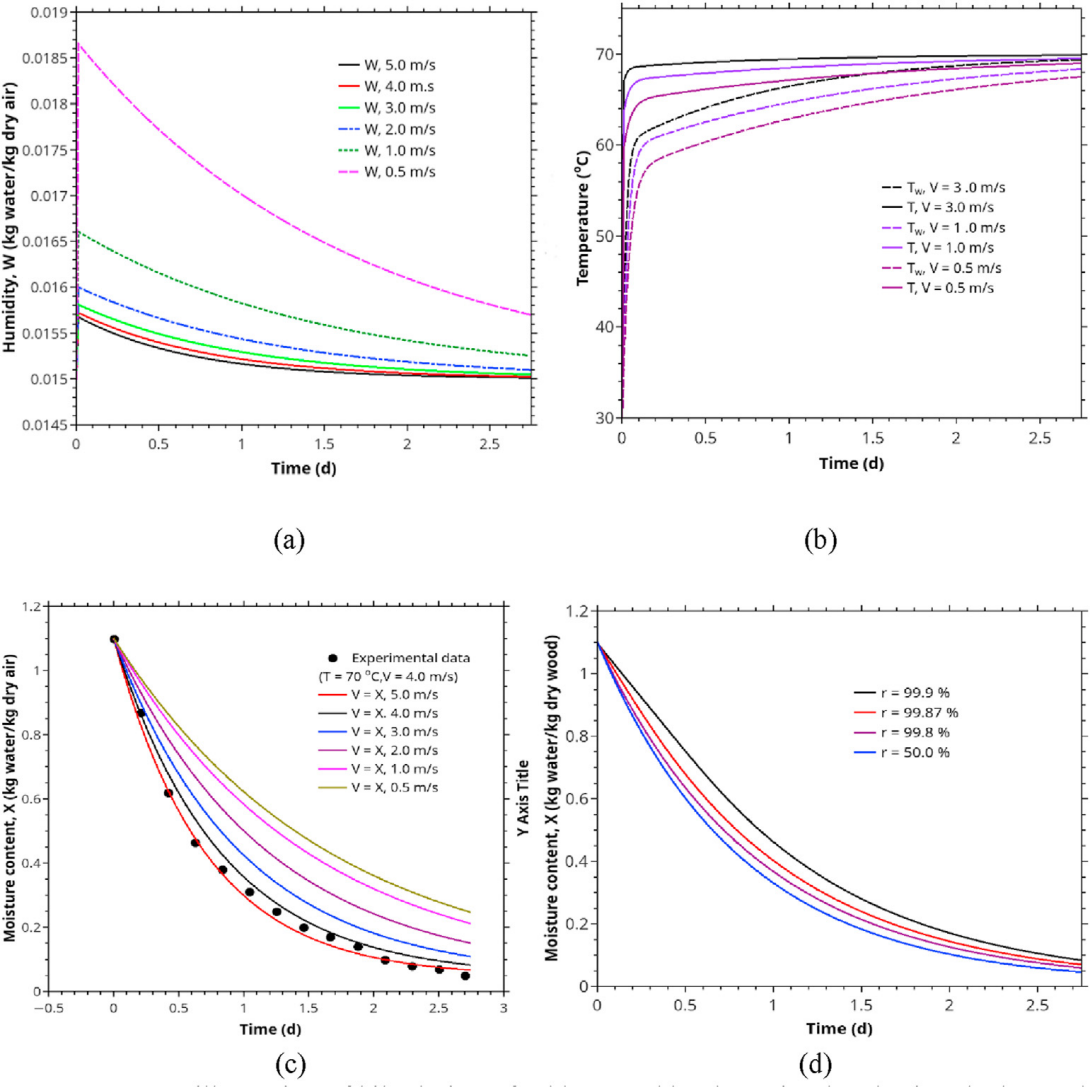
An illustration of kiln drying of rubberwood lumbers simulated using the lumped parameter drying model: (a) air humidity versus time for different humidity of inlet air, (b) wood and air temperature versus time at 0.5, 1.5, 3.0 m/s and 70 °C, (c) comparison between simulation and actual kiln drying data [11], (d) the effect of air recycle ratio on the drying curves. We chose the velocity of 0.5 m/s because, at a higher velocity, the recycling ratio's effect becomes less significant.

In Figure 14, we also illustrate a sample of drying curve prediction for industrial kiln drier. In generating this prediction, we set the model parameters to typical values and summarized them in Table 6. The stack moisture transfer area (A) was calculated from each lumber's main dimensions (2 sides x1m x 0.08m x 100 items, subtracted by 10% occupied by separating sticks). The mass of dry wood ($M_{0}$) was calculated from the initial lumbers' mass subtracted by the initial moisture content. The dry air mass in the kiln drier was calculated by the total air space in the kiln drier (2.6 m^3^) and the air density at 70 °C (1.029 kg/m^3^). The air velocity and the recycle ratio were taken directly from Tomad et al. [11]. The heat transfer coefficient (*h*) was chosen from the literature for air velocity in the range of 2.5–5.0 m/s and 56 °C by observing no significant change in *h* over the temperature range of 50–80 °C [72]. Specific heat of dry wood was obtained from Radmanović et al. [77]. The other parameters were calculated from psychrometry. The model (Eqs. (18), (19), (20), (21), (22), (23), (24), and (25)) calculated the corresponding parameters from these parameters according to wood/air moisture content and temperature.

**Table 6:** Process parameters used in the simulation of the lumped-parameter drying model.

Parameters	Symbol	Measured/Estimated	Unit
Stack transfer area	A	15.3	(m^2^)
Mass of dried wood	$M_{0}$	135.29	(kg)
Mass of dry air in the kiln drier	$M_{a}$	2.675	(kg)
Air velocity	V	0.5, 1.0, 2.0, 3.0, 4.0, 5.0	(m/s)
Heat transfer coefficient, for V = 0.5, 1.0, 2.0, 3.0, 4.0, 5.0 m/s respectively. Source/interpolated: Trembay et al. [82]	h	19.9, 23.0, 28.0, 31.5, 33.5, 34.0	(Wm^−2^K^−1^)
Air recycle ratio (*T* = 70 °C, V = 4.0 m/s and corresponding to *G*_0_ = 2.058, 0.0082, 0.0054, 0.0041 kg/s, respectively)	r	50.00, 99.80, 99.87, 99.09	(%)
Mass air-flow rate before mixed with recycling air for V = 0.5, 1.0, 2.0, 3.0, 4.0, 5.0 m/s respectively.	$G_{0}$	0.343, 0.686, 1.715, 2.401, 3.430	(kg s^−1^)
Mass air-flow rate after mixed with recycling air for V = 0.5, 1.0, 2.0, 3.0, 4.0, 5.0 m/s respectively	G	0.515, 1.029, 2.573, 3.601, 5.145 (for *r* = 50%)	(kg s^−1^)
Latent heat of vaporization at 0 °C	$\Delta h_{0}$	2.501e6	(Jkg^−1^)
Latent heat of vaporization at a drying temperature	$\Delta h_{v}$	2.333e6	(Jkg^−1^)
Specific heat of dry wood at a drying temperature	$c_{ps}$	1,360	(Jkg^−1^K)
Specific heat of the water in wood at drying temperature	$c_{pl}$	4216	(Jkg^−1^K)
Specific heat of water vapor at a drying temperature	$c_{pv}$	1880	(Jkg^−1^K)
Specific heat of air at 70 °C	$c_{pa}$	1783.62	(Jkg^−1^K)
Air inlet temperature	$T_{in}$	70	(^o^C)
The moisture content of the air inside the kiln at the initial time	$W_{0}$	0.015	(kg water/kg dry air)
The humidity of entering air before mixed with recycling air	$W_{a0}$	0.015	(kg water/kg dry air)
Initial moisture content in wood	$X_{0}$	1.10	(kg water/kg dry wood)
The equilibrium moisture content of wood which depends on air temperature and relative humidity	$X^{*}$	0.033	(kg water/kg dry wood)
The initial temperature of the air in the kiln dryer	$T_{0}$	30	(^o^C)
The initial temperature of wood in the kiln dryer	$T_{w0}$	30	(^o^C)
Specific enthalpy of entering air before mixed with recycling air	$H_{a0}$	89,349.6	(J/kg dry air)

In general, the model suggests that it is possible to complete the drying process within two days of drying operation. As for the current industrial practice for kiln drying of rubberwood lumbers, the manufacturers mostly used 5–7 days for drying stacked rubberwood lumbers. Considerable time and energy-saving are possible, and we are currently exploring it. However, this conclusion is applied for the drying rate but not for ensuring high product quality, which we will not discuss further.

**Figure 15:**
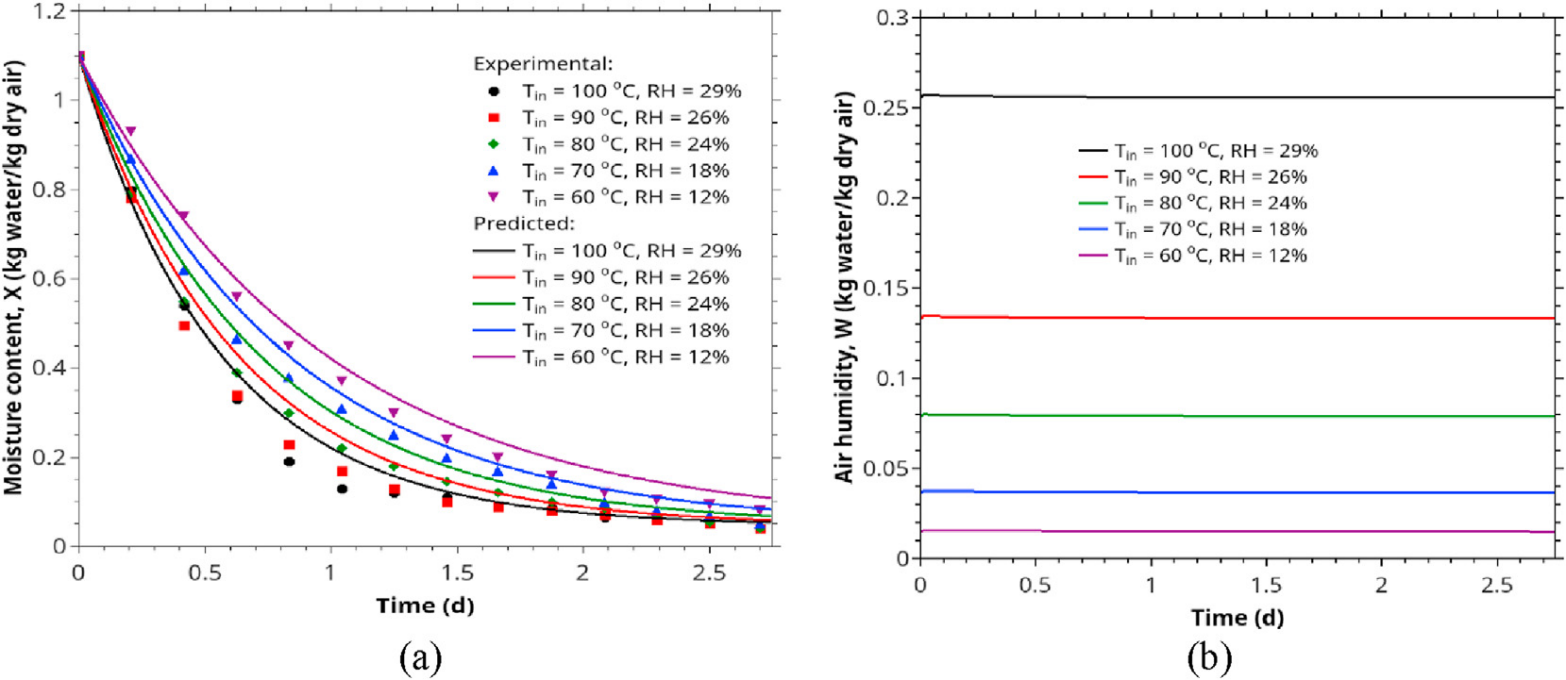
The effect of temperature and RH on the moisture content in wood and air humidity. EMC is fixed at 3.3%, %RH of inlet air was controlled as 12, 18, 24, 26, 29 (or 15.11, 36.65, 79.04, 133.27, 255.80 g/kg dry air) for air temperature of 60, 70, 80, 90, and 100 °C respectively.

Figures 14 and 15 illustrate how to use the correlation developed in this work for kiln drying simulation. This simulation is based on a lumped-parameter drying model that assumes well-mixed airflow in the drying chamber. However, the overall mass transfer coefficient used in the simulation is based on the average value for the stacked lumbers, compensated for non-ideal mixing. Hence, with limited data currently available to validate the model, we presented only five sets of actual data, as shown in Figures 14(c) and 15(a), which agree well with the simulation results, albeit the model underpredict the drying rate in the initial phase of drying. The discrepancies are mainly due to the CRP's negligence of CRP in developing the correlation for the overall mass transfer coefficient. Thus, for better accuracy, one must incorporate the kinetics of drying in the initial phase (CRP) in the correlation. More intensive data from actual rubberwood kiln-drying will be required to validate this model in practice. However, we hope this illustration will serve the purpose of this article.

For our cases, the model predicts that lumbers' temperature lacks behind that of the bulk air by 5–10 °C at the early hours (Figure 14b). The lacking reduces to approximately 5 °C at 24 h. If the inlet air temperature is 70 °C and V = 3.0 m/s, the lumber temperature starts at about 60 °C initially and reaches 65 °C after 24 h. However, the air temperature reaches 65 °C in a few hours of drying. Some researchers report that the overall heat transfer coefficient is sensitive to lumbers' surface temperature in the range of 50–80 °C [72]. However, regardless of the mechanisms behind the moisture transfer, according to our experimental data, it is evident that the drying kinetics of rubberwood lumbers (MR vs. time) in FRP follows first-order kinetics (Eq. (9)). The fact implies that FRP's overall moisture transfer coefficient is nearly constant over this work's experimental range. The result agrees with the observations of Ananias et al. [64,65] and Chrusciel et al. [48] for other woods. Thus, in FRP, it is sufficient to correlate *K* with air temperature without considering the dependent dynamic of the simultaneous heat and mass transfer. The prediction in Figure 14 (b) is based on the enthalpy balance. Since *K* is approximately constant, while the lumbers' temperature is low (so too the moisture diffusivity inside the wood, water vapor at the interface, and the specific heat of the wood) is compensated by a relatively high-temperature gradient ($T-T_{w}$). The overall result is the appearance of constant overall heat and mass transfer coefficients. To some extent, this result validates that Ananias' approach is applicable in the conventional rubberwood kiln drying.

In general, the model suggests that it is possible to complete the drying process within 2 days of drying operation with a suitable operating profile. However, this conclusion is applied for drying rate and drying time but not for ensuring high product quality. Thus, to use model prediction developed here in practice, one must consider both energy minimization criteria, the drying profile (temperature, air velocity, and RH versus time), and the standard practice for high-quality products.

With this simple lumped parameter model and our correlation, it is possible to predict the effect of air velocity, drying temperature, RH, and air-recycle ratio on the drying process's performance subjected to various dynamic conditions. If sufficient operating data are available for calibrating the models, it is expected that it can be used for process control and optimization in the future. For now, the authors could claim that the correlation for the overall mass transfer coefficient developed in this work can be used for process simulation of kiln drying of stacked rubberwood lumbers in the experimental range cover in this work. More future work may be needed to refine the correlation for more extensive conditions and accuracy, however.

## 4 Conclusion

This work has fulfilled the much-needed correlation for estimating the rubberwood lumber drying's overall moisture transfer coefficient in stacked kiln driers. The correlation is based on the modification of the Ananias formula. Its operational range of applicability is 60–100 °C, 0.5–4 m/s air velocity, 6–67%RH, and 20–30 mm lumber thickness, which covers most industrial practices for rubberwood drying in Thailand. It thus forms a basis for kiln drying process design, monitoring, control, and optimization for both future research and industrial practices. Our correlation provides an essential tool to estimate the design and operational parameters that are accurate and fundamental enough to represent wood lumber's behavior in response to different flow and psychometric environments. This work also confirms that, in practical terms, the conventional theory of drying can sufficiently describe the drying mechanisms of rubberwood. Furthermore, it is possible and sufficient to characterize the drying kinetic in constant and falling rate period with five parameters (and other physical wood characteristics), namely: evaporation rate in constant rate period, overall mass transfer coefficient in falling rate period, critical moisture content, fiber saturation point, and EMC.

This work contributes to the understanding of the drying kinetics of rubberwood lumbers. It also provides a simple method for prediction by using a lumped-parameter drying model. It also suggests that for the lumbers having the dimension of 1 m length × 8 cm width × 3 cm thickness, and the air velocity >1.5 m/s, it is possible to achieve the final dried product within 2.5 days. As the normal drying cycle currently used in most factories is more than four days. Well-design and proper drying operation could substantially reduce the drying time and energy cost.

There are two more contributions to be mentioned. Firstly, it was possible to represent all drying curves covering both periods using combined drying kinetic model with the help of a suitable switching function. Secondly, this work clearly shows that kinetic studies of stacked rubberwood lumber drying require more careful experimental design and a more intensive analysis of the results to be directly applicable for engineering practices. More relevant data and analysis to reinforce the current work in the future should be welcome. Different approaches, such as Weibull and Bi-Di models, should be further explored to enhance the understanding and obtain more advanced engineering practices.

## Declaration

### Author contribution statement

Chairat Siripatana: Conceived and designed the experiments; Analyzed and interpreted the data; Wrote the paper.

Malisa Chanpet: Performed the experiments; Analyzed and interpreted the data.

Nirundorn Matan & Nirattisai Rakmak: Contributed reagents, materials, analysis tools or data.

### Funding statement

This work was supported by the 10.13039/501100010034Walailak University Fund (Contact no. 11/59), Biomass and Oil-Palm Excellence Center Walailak University, Thailand.

### Competing interest statement

The authors declare no conflict of interest.

### Additional information

No additional information is available for this paper.
